# Micronutrient status of COVID-19 patients: a critical consideration

**DOI:** 10.1186/s13054-020-03085-0

**Published:** 2020-06-16

**Authors:** Anitra C. Carr

**Affiliations:** Nutrition in Medicine Research Group, Department of Pathology & Biomedical Science, University of Otago, Christchurch, PO Box 4345, Christchurch, 8140 New Zealand

As the novel coronavirus (SARS-CoV-2) pandemic continues to grip the world, ongoing research is highlighting the factors associated with higher risk of severe morbidity and mortality. These factors include older age, male, and comorbidities, including obesity [1]. Relatively little attention, however, is paid to the micronutrient status of COVID-19 patients. Micronutrients are vitamins and specific minerals that are critical to the proper structure and functioning of numerous proteins, enzymes, physiological processes, and signaling pathways within the body. Without these micronutrients, these essential processes cease to function properly, and this contributes to morbidity and, in cases of severe deficiency, mortality.

The term “micronutrients” refers to the fact that these nutrients are required in small, usually microgram, amounts daily. However, in critical illness, the requirements for these micronutrients can increase significantly. This is particularly true for vitamin C, which is severely depleted in critically ill patients at admission and is required in gram amounts to replete the patients [2]. Severe respiratory infections, such as pneumonia, are a common complication of severe vitamin C deficiency, and pneumonia is one of the most common causes of mortality in patients with severe vitamin C deficiency [3]. This indicates an important link between vitamin C status and respiratory infections.

Vitamin C has numerous important and pleiotropic roles in immune function, including regulation of hundreds of genes in immune cells [3]. Although the vitamin C status of patients with COVID-19 has not yet been reported in the literature, it is known that the vitamin C status of patients with community-acquired pneumonia is severely depleted and is associated with enhanced oxidative stress [4]. However, it is not yet known if vitamin C deficiency is a cause and/or a consequence of severe infection, as the latter results in enhanced requirements for the vitamin.

There are numerous factors, in addition to inadequate diet, that are associated with depleted vitamin C status [5]; these include male gender, comorbidities, and obesity, which are also associated with enhanced susceptibility to COVID-19. Therefore, it is important to consider that differences in susceptibility to, and severity of, COVID-19 could be partly due to insufficient micronutrient levels for adequate immune and organ function. As such, it would be of benefit to COVID-19 patients if critical care physicians take this into consideration and test for potential micronutrient insufficiencies in their patients and, if indicated, supplement with adequate amounts to restore normal status and function. This may help improve patient outcomes.

## Data Availability

N/A
